# A Rare Presentation of Community Acquired Methicillin Resistant *Staphylococcus aureus*

**DOI:** 10.1155/2013/543762

**Published:** 2013-12-28

**Authors:** J. Docekal, J. Hall, B. Reese, J. Jones, T. Ferguson

**Affiliations:** ^1^Department of Internal Medicine, Tripler Army Medical Center, 1 Jarrett White Road, Honolulu, HI 96859, USA; ^2^Tripler Army Medical Center, 1 Jarrett White Road, Honolulu, HI 96859, USA

## Abstract

Prostatic abscess is a rarely described condition and is commonly caused by gram-negative organisms such as enterobacteria. However, as the prevalence of methicillin resistant *Staphylococcus aureus* (MRSA) increases in the community, unusual infections due to this organism have been recently published. In this report, we describe a patient with diabetes mellitus type 2, who presents with diabetic ketoacidosis—later found to be due to a prostatic abscess from which MRSA was cultured.

## 1. Introduction

Prostatic abscess is an uncommon condition that has been associated with the presence of chronic indwelling catheters, genitourinary instrumentation, diabetes mellitus, acquired immunodeficiency syndrome, hemodialysis, or other immune compromising conditions [1].

Prior to the widespread use of broad spectrum antibiotics for patients with lower urinary tract infections, this condition was primarily caused by *Neisseria gonorrhoeae*. Currently, *E. coli* and other gram negative bacteria are the primary pathogens responsible for the development of prostatic abscesses [1].

As the incidence of community acquired methicillin resistant *Staphylococcus aureus* (CA-MRSA) increases, there has been an emergence of published case reports regarding the development of MRSA prostatic abscesses. While these cases present an exceedingly rare site for invasive MRSA infection, we feel that, with the increasing prevalence of community acquired MRSA, there is increased future potential for MRSA prostatic abscesses. Consequently, practitioners should develop a high suspicion for this condition, particularly when an immunocompromised patient presents with lower urinary tract symptoms.

## 2. Case Discussion

A 56-year-old male resident of a homeless shelter—with a past medical history significant for poorly controlled type 2 insulin-dependent diabetes mellitus—presented to the emergency department with complaints of dysuria, polyuria, polydipsia, and difficulty urinating. He was found to be hyperglycemic, and other laboratory findings were consistent with the diagnosis of diabetic ketoacidosis (DKA). The patient was subsequently admitted to the ICU and was treated effectively for DKA with resolution within 12 hours, at which time he was transferred to the medical floor for further management.

Approximately two days prior to admission, this patient was seen at his outpatient clinic with complaints of dysuria and was empirically treated with oral ciprofloxacin; however, his symptoms persisted and worsened. A urine culture was sent at the time of initial presentation, which eventually returned with no growth.

When the patient was transferred to the medicine service, he was interviewed and examined. He endorsed rectal pain, obstipation, and continued urinary retention. On physical exam, he exhibited lower extremity edema, 2+ pitting, and had a few scattered nonblanching erythematous macular rashes on his thighs and upper extremities. His rectal exam revealed no fluctuance, mass, or fissures, but an enlarged and tender prostate was noted.

**Figure 1:**
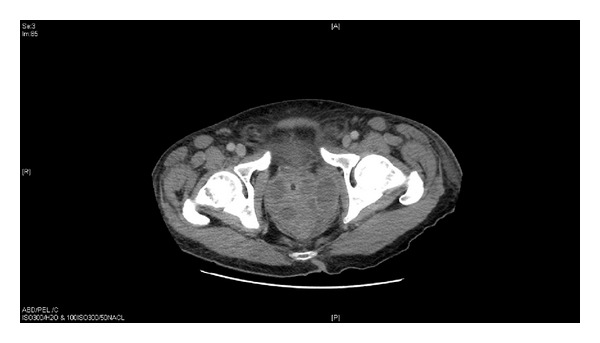
There is a multiloculated fluid collection centered in the prostate gland with multiple enhancing septations. Overall this lesion measures approximately 9 × 8 × 8 cm.

A sexual history was obtained but was unrevealing, and additional labs were obtained to evaluate for sexually transmitted diseases, including HIV. His physical exam findings were a concern, prompting the medicine team to obtain a CT of his abdomen and pelvis, ultimately discovering a 9-10 cm prostatic abscess (see Figure 1), the likely culprit for etiology of his diabetic ketoacidosis.

Interventional radiology and urology were both consulted shortly after the CT results, and the patient then underwent an IR-guided abscess drainage. Cultures from the abscess were positive for MRSA, and treatment with vancomycin was started.

Following the initial drainage procedure, subsequent imaging noted reaccumulation of the prostatic abscess, which necessitated a second IR drainage procedure and placement of two 8Fr pigtail catheter drains. Approximately two weeks after pigtail drain placement, abscess drainage continued at an unacceptable rate. Accordingly, the patient underwent a transurethral resection of the prostrate (TURP), with unroofing of the prostatic abscess. Following the TURP procedure, the patient's inflammatory markers and serum prostate specific antigen were noted to consistently downtrend. The patient continued to improve and was discharged with a plan to complete 6 weeks of vancomycin therapy.

## 3. Discussion

The development of diabetic ketoacidosis has long been associated with intercurrent illnesses, such as infection, inadequate insulin therapy, ischemia, inflammation, and newly diagnosed diabetes. Interestingly, it is estimated that infection is present in 48% of all type 2 diabetics in DKA, as compared to that in 22% of patients with type 1 diabetes in DKA [2]. This case is unique in that a presentation of diabetic ketoacidosis was precipitated by an unusual infectious source and an even more uncommon presentation of CA-MRSA.

The clinical spectrum of community acquired MRSA infection includes asymptomatic colonization, skin and soft tissue infections (SSTI), and invasive infections. MRSA SSTI present as furuncles, carbuncles, or superficial abscesses [3]. Invasive infections are initiated when a breach of the skin or mucosal barrier allows staphylococci access to the blood stream or adjacent tissues [4]. When associated with invasive infection, MRSA may present in a variety of manners, such as endocarditis, internal abscesses, necrotizing pneumonia, myositis, fasciitis, or sepsis syndrome.

The diagnosis of prostatic abscess is clinically challenging. This is likely due to the nonspecific clinical signs and the rarity of this condition, particularly in the current era of widespread antibiotic use for lower urinary tract infections. Typical signs and symptoms include urinary hesitancy, weakened stream, rectal pain, constipation, low back pain, dysuria, leukocytosis, and systemic signs of infection.

Known risk factors for prostatic abscess include indwelling catheters, prostrate biopsy, immune compromising medical condition, and instrumentation of the lower urinary tract. As with the patient in this report, the most common cause of immunodeficiency among patients diagnosed with MRSA abscess of the prostrate was poorly controlled diabetes mellitus.

We noted that following the patient's unroofing procedure, there was a parallel decrease in serum prostate specific antigen (PSA), erythrocyte sedimentation rate, and C-reactive protein. These down trending markers correlated with progressive patient clinical improvement. We feel that this is an interesting and noteworthy observation, which suggests that the serum PSA may be used as a marker for monitoring response to therapy, during treatment for prostatic abscess (Table 1).

In conclusion, we report a CA-MRSA prostatic abscess as the underlying culprit for DKA in a homeless male with poorly controlled type 2 diabetes mellitus. This case highlights the need for increased vigilance among physicians for atypical presentations of invasive infection with MRSA. Furthermore, we suggest that there is potential utility in serial measurements of serum PSA levels, as a guide to predict treatment response.

## Figures and Tables

**Table 1:** 

	ESR	C-reactive protein	PSA serum
June 20th	94	7.2	4.26
June 22nd		6.9	
June 28th		1.67	
July 3rd		0.85	
July 5th	71	2.3	2.75
July 12th	46	0.56	1.32
July 19th	39	<0.5	1.31
July 26th	27	<0.5	0.76
